# 2013 Korean Society of Hypertension guidelines for the management of hypertension: part III-hypertension in special situations

**DOI:** 10.1186/s40885-014-0014-1

**Published:** 2015-04-15

**Authors:** Jinho Shin, Jeong Bae Park, Kwang-il Kim, Ju Han Kim, Dong Heon Yang, Wook Bum Pyun, Young Gweon Kim, Gheun-Ho Kim, Shung Chull Chae, The Guideline Committee of the Korean Society of Hypertension

**Affiliations:** Department of Internal Medicine, Hanyang University College of Medicine, Seoul, Korea; Division of Cardiology, Department of Medicine, Cheil General Hospital, Dankook University College of Medicine, Seoul, Korea; Department of Internal Medicine, School of Medicine, Seoul National University, Bundang, Korea; Department of Internal Medicine, School of Medicine, Chonnam University, GwangJu, Korea; Division of Cardiology, Department of Internal Medicine, Kyungpook National University School of Medicine, 130 Dongdeok-ro, Jung-gu, Daegu, 700-721 Korea; Division of Cardiology, Department of Internal Medicine, Ewha Womans University School of Medicine, Seoul, Korea; Division of Cardiology, Department of Internal Medicine, Dongkuk University College of Medicine, Ilsan, Korea

**Keywords:** Antihypertensive treatment, Guidelines, Hypertension, Complications

## Abstract

Different treatment strategies are suggested for special situations. Hypertension is common in the elderly and frequently accompanied by or complicates other clinical conditions such as metabolic syndrome, coronary artery disease, heart failure, stroke, diabetes mellitus and chronic kidney disease.

## Hypertension in special situations

### White coat hypertension and masked hypertension

Emphasizing out-of-office BP measurements, white coat hypertension (HTN) and masked HTN are acknowledged as new categories of BP by performing ABPM or home BP monitoring. Although the clinical evidence is not established, some expert opinions exist for these two categories. For most subjects with white coat HTN, lifestyle modifications and regular BP monitoring are recommended. Despite the weak evidence, when metabolic disturbance and/or subclinical organ damage occur with white coat HTN, pharmacologic treatment as well as lifestyle modifications could be considered [1,2]. Strict pharmacologic treatment for masked HTN may be beneficial because it showed a similar cardiovascular (CV) risk profile to that of sustained HTN [3].

### Metabolic syndrome

Many hypertensive patients have obesity and metabolic abnormalities with alterations of lipid and glucose metabolism. Furthermore, subclinical organ damage is not uncommon in these patients. Metabolic syndrome involves abdominal obesity, dyslipidemia, dysglycemia, and raised BP. The criteria for clinical diagnosis of metabolic syndrome are 1) abdominal obesity; 2) fasting glucose ≥100 mg/dL (diabetes included); 3) triglyceride ≥150 mg/dL; 4) HDL cholesterol <40 mg/dL in men and <50 mg/dL in women; and 5) BP ≥130/85 mm Hg. Presence of three or more of these criteria confirms the diagnosis of metabolic syndrome [4]. Abdominal obesity is usually estimated by the measurement of waist circumference. However, cut points of waist circumference for abdominal obesity in Korean adults are not established. Cut points of waist circumference, which are commonly used, are 1) >90 cm in men; >80 cm in women (International Obesity Task Force criteria for Asian-Pacific population) [5] or 2) ≥90 cm in men; ≥85 cm in women (Korean adult specific values) [6].

The prevalence of metabolic syndrome has been increasing for the past 10 years, as reported in the Korean National Health and Nutritional Survey. Twenty-five to thirty percent of Koreans over 20 years have metabolic syndrome. However, the prevalence of metabolic syndrome is much higher in hypertensive patients, reaching two times that of the adult population [7].

In Western countries, people with metabolic syndrome are at 1.5 to 2 times higher risk of CV events and death than those without metabolic syndrome, [8] and incident diabetes is five times higher in people with metabolic syndrome [9]. In Asian countries, metabolic syndrome had a relative risk of incident diabetes of three to four, which is a little lower compared to that in Western countries [10,11]. The most powerful predictor of incident diabetes is hyperglycemia in people with metabolic syndrome. However, metabolic syndrome, even without hyperglycemia, was associated with an increased risk of incident diabetes; the relative risk is 2.4 in Japanese populations [11]. In addition to metabolic syndrome, HTN is a risk factor for incident diabetes, [12] with a relative risk for incident diabetes of two [13,14].

In hypertensive patients with metabolic syndrome, antihypertensive treatment aims mainly to prevent CV morbidity and mortality, while lowering or preventing incident diabetes. Antihypertensive treatment in non-diabetic patients with metabolic syndrome is discussed below, while that in diabetic/cardiovascular disease (CVD) patients with metabolic syndrome are discussed in other respective sections of the special situations chapter. Lifestyle modifications, especially weight reduction and regular exercise, are strongly recommended in all hypertensive patients, as they decrease BP, improve metabolic abnormalities, and delay incident diabetes.

Antihypertensive drugs to be selected should have adequate BP lowering efficacy, as well as favorable or neutral effects on insulin sensitivity and metabolic abnormalities. Thus, angiotensin-converting enzyme ACE inhibitors, angiotensin receptor blockers (ARBs), and calcium antagonists are preferred. Among beta-blockers, vasodilating beta-blockers such as carvedilol and nebivolol can be selected when angiotensin-converting enzyme inhibitors or angiotensin receptor blockers are avoided. Carvedilol and nebivolol have no unfavorable effects on glucose and lipid metabolism [15-17]. These beta-blockers can be used as combination therapy with angiotensin-converting enzyme inhibitors or angiotensin receptor blockers. However, the BP lowering efficacy is lower with a combination of beta-blockers and angiotensin-converting enzyme inhibitors or angiotensin receptor blockers than with a combination of beta-blockers and calcium antagonists. Old generation beta-blockers such as propranolol, atenolol, and metoprolol are associated with an increased risk of new onset diabetes and unfavorable effects on lipid metabolism [18,19]. The long-term use of these drugs as monotherapy needs to consider the risk for new onset diabetes and dyslipidemia.

Thiazides and thiazide-like diuretics are avoided as monotherapy or at high dose but used as combination therapy or at low dose. These diuretics can induce hypokalemia and new onset diabetes and have unfavorable effects on lipid metabolism. Combination with old generation beta-blockers is avoided. They can be used with potassium-sparing diuretics. The preferred approach is combination with angiotensin-converting enzyme inhibitors and angiotensin receptor blockers to minimize their unfavorable effects on glucose and lipid metabolism.

The combination of angiotensin-converting enzyme inhibitors and angiotensin receptor blockers and thiazide diuretics is less favorable in terms of CV events and incident diabetes than combination of angiotensin-converting enzyme inhibitors or angiotensin receptor blockers and calcium antagonists [20].

### Diabetes mellitus

The prevalence of HTN is twofold in diabetic patients compared to that in the general population, and the occurrence of diabetes is 2.5 times higher in hypertensive patients [14,19]. The coexistence of HTN and diabetes causes the progression of CVD, stroke, and renal disease. The high risk of HTN in diabetic patients is known to be due to weight gain and hyperinsulinemia, hyperactivity of sympathetic nervous system, and the increase of body fluids by the renal retention of sodium. Additionally, hyperglycemia further increases the risk of HTN by increasing arterial stiffness and progressing atherosclerosis. The nocturnal dipping disappears in diabetic patients, and it is related with subclinical organ damage such as left ventricular hypertrophy (LVH) and microalbuminuria. In UKPDS-36, each 10 mm Hg decrease in mean systolic blood pressure (SBP) was associated with risk reductions of 12% for any complication related to diabetes, 15% for deaths related to diabetes, 11% for myocardial infarction, and 13% for microvascular complications [21]. Previous studies show that appropriate BP control can reduce the incidence of CVD [22-25].

The recommended target for BP in diabetic patients is <140/85 mm Hg. In previous guidelines, the recommended BP was <130/80 mm Hg or <140/80 mm Hg in diabetic patients [26]. However, recent studies showed that maintaining a low BP did not result in a reduction of the incidence of CV events [27]. Therefore, the cost-effectiveness and side effects of antihypertensive drugs should be considered when the BP drops excessively in diabetic patients.

According to a recent meta-analysis, all classes of antihypertensive agents, such as ACE inhibitors, ARBs, calcium channel blockers, beta-blockers, and diuretics, are useful [28]. ACE inhibitors and ARBs are recommended as first line antihypertensive therapy in a patient without compelling indications. The superiority of one antihypertensive class over others is controversial. The choice of a particular class is less significant in practice because two or more antihypertensives should be combined in order to obtain sufficient decrease in BP in most diabetic patients. However, the combination of beta-blockers and thiazide diuretics needs to be prescribed with caution as it could worsen glucose control by increasing insulin resistance [18].

### Hypertension in older adults

The treatment of HTN in older adults reduces the occurrence of vascular disease and mortality. The benefit of treatment is also observed with regard to isolated systolic HTN. Accordingly, HTN needs to be actively diagnosed and treated in older adults [29].

However, the pharmacological treatment of stage I HTN in patients aged 80 years or older remains undetermined. Thus, the characteristics of the patient should be considered. The characteristic findings in elderly hypertensive patients are increased SBP and pulse pressure due to increased central arterial stiffness. Moreover, atherosclerotic renovascular HTN is commonly observed. Non-dipper, increased daytime BP variability, and orthostatic or postprandial hypotension are also characteristic findings in elderly patients with HTN.

The non-pharmacological treatment in elderly HTN patients is effective; however, the impact on patients’ quality of life should be considered [30]. The target SBP for older patients is <140 to 150 mm Hg, but orthostatic hypotension should be avoided [31,32]. Additional studies are required to confirm the target SBP in old or frail patients.

The initial dose of the pharmacologic treatment is reduced by half in younger patients and gradually increased. Elderly hypertensive patients without comorbidities should be treated with ACE inhibitors, angiotensin receptor blockers, calcium antagonists, and diuretics [33-36]. Beta-blockers do not improve prognosis as much as other drug classes in elderly hypertensive patients [37-39]. However, beta-blockers would be effective in patients with angina, heart failure, or tachycardia. Combination therapy with two or more drugs should be considered if the BP is not controlled with monotherapy. Patients with comorbidities require special consideration. It is safe to slowly lower the BP in elderly patients. Complications caused by medication should be monitored when increasing the drug dose. Orthostatic hypotension should be periodically checked by positional BP measurement.

### Cardiovascular diseases

#### Coronary artery disease

HTN is a major risk factor of coronary artery disease and is associated with the occurrence of myocardial infarction [25]. The incidence of ischemic heart disease increases when SBP is >140 mm Hg [40] and mortality increases when SBP is >120 mm Hg [41,42]. Previous guidelines have recommended a target SBP <130 mm Hg in patients with coronary artery disease, although the level of evidence is weak. Therefore, the present recommended SBP in coronary artery disease is <140 mm Hg.

The preferred drugs within 1 month after acute myocardial infarction are beta-blockers [43] and angiotensin-converting enzyme inhibitors [44]. Any first line antihypertensive drugs are available in other types of ischemic heart disease. In the case of symptomatic coronary artery diseases, beta-blockers and calcium antagonists need to be considered first.

#### Chronic heart failure

HTN is the most important risk factor in heart failure [45]. Most BP lowering drugs such as diuretics, beta-blockers, ACE inhibitors, and angiotensin receptor blockers are effective in the prevention of heart failure [46]. Low BP in patients with systolic dysfunction and HTN is associated with poor CV outcomes, despite high BP being a risk factor for heart failure [47]. Few studies have investigated patients with heart failure and low BP because most prospective randomized studies did not include patients with low BP. The appropriate BP is determined based on the patient's situation. However, indirect studies showed that beta-blockers, ACE inhibitors, ARBs, and aldosterone antagonists are useful for modifying the harmful effects of the sympathetic nervous system and renin-angiotensin-aldosterone system and are the preferred drugs in patients with heart failure [48]. HTN is also a risk factor for heart failure with preserved left ventricular systolic function. Most studies in patients with preserved heart failure revealed no additional benefits from lowering the SBP below 140 mm Hg [49].

#### Atrial fibrillation

Atrial fibrillation is frequently observed [50] and preventable with BP control in patients with HTN [51]. Patients with HTN and atrial fibrillation have a high risk of thromboembolism and need chronic antithrombotic treatment if no contraindications are present [52]. Recently, antithrombotics such as the thrombin inhibitors dabigatran, factor Xa inhibitors, rivaroxaban, and apixaban have been shown to be more effective and relatively safe compared with the classic established therapy using warfarin [53]. In patients with atrial fibrillation and HTN, lowering the BP can decrease the incidence of fatal bleeding during antithrombotic treatment [54]. Beta-blockers and non-dihydropyridine calcium antagonists are useful for controlling the heart rate. In patients with HTN and LVH, ACE inhibitors or ARBs are effective for primary prevention of atrial fibrillation [55-59]. However, the benefits of ACE inhibitors are negligible in patients with chronic atrial fibrillation or known atherosclerosis [60-65]. Beta-blockers and aldosterone antagonists are useful for the prevention of atrial fibrillation in patients with heart failure [65,66].

### Other arterial diseases

#### Carotid atherosclerosis

The progression of carotid atherosclerosis is decreased by lowering the BP. For this purpose, calcium antagonists and ACE inhibitors are superior to beta-blockers and diuretics [67,68].

#### Arterial stiffness

Most antihypertensive drugs decrease vascular stiffness because lowering the BP decreases vascular wall stress and pulse wave velocity. Other than the BP lowering effect, inhibitors of the renin-angiotensin-aldosterone system decrease the pulse wave velocity, regardless of BP, [69-71] while vasodilating beta-blockers decrease the central aortic SBP compared to that with atenolol [72]. Although an improvement in vascular stiffness with antihypertensive drugs has been reported in numerous studies, it is still uncertain whether the improvement in vascular stiffness is closely related to the CV benefits, except for patients with specific condition [73]. Further studies are needed to determine the relationship between vascular stiffness and CV outcomes.

#### Peripheral artery disease

It is important to control the CVD risk factors because patients with peripheral artery disease have a higher risk of CV mortality (10-year mortality of 40%) [74]. Lowering the SBP decreases the leg amputation rate and mortality in hypertensive patients with diabetes and peripheral artery disease. The target BP is <140/90 mm Hg in patients with peripheral artery disease.

Lifestyle modifications such as salt restriction, weight control, moderation of alcohol intake, and regular aerobic exercise are very important. Pharmacologic treatment consists of ACE inhibitors, ARBs, and aspirin. ACE inhibitors decrease long-term CV events from either BP lowering effects or indirect BP lowering effects [25,75]. However, other drugs are also effective in the reduction of CV events associated with BP lowering [25]. Furthermore, it is important to evaluate and manage CV risk factors other than HTN, such as lipid and blood sugar. The appropriate drugs are determined according to the presence of heart failure or coronary artery disease. Generally, beta-blockers are relatively contraindicated to avoid worsening of peripheral artery disease symptoms. However, some reports revealed that beta-blockers do not increase the symptoms in patients with mild to moderate peripheral artery disease severity. Therefore, beta-blockers are effective in peripheral artery disease patients with coexisting ischemic heart disease or tachycardia [76-78]. Renal artery stenosis is frequently observed in patients with HTN and peripheral artery disease. Overall, ongoing evaluation of the disease and monitoring are needed during HTN treatment [79].

### Chronic kidney disease

Chronic kidney disease (CKD) is defined by the presence of kidney injury for ≥3 months, with the markers of kidney injury being a decrease in estimated glomerular filtration rate (<60 mL/min/1.73 m^2^), urinary abnormalities including albuminuria (≥30 mg/day or albumin-to-creatinine ratio ≥30 mg/g), hematuria and pyuria, electrolyte disturbances caused by tubular dysfunction, renal structural abnormalities detected by imaging or biopsy procedures, and renal transplants [80]. CKD patients frequently suffer from HTN; hence, the rate of decline in renal function and the incidence of CV complications can be reduced with HTN control [81,82]. However, we still need to determine the target BP levels, optimal tools to be used in HTN control, and the real benefits and risks associated with treatment [83].

Previous clinical practice guidelines including the seventh report of the Joint National Committee on the Prevention, Detection, Evaluation, and Treatment of High Blood Pressure (JNC7) and Kidney Disease Outcomes Quality Initiative (KDOQI) recommended a BP target of <130/80 mm Hg in all CKD patients [84,85]. However, recent major clinical trials failed to show that in non-proteinuric CKD patients, a strict BP target of <125/75 to <130/80 mm Hg is more beneficial than a conventional target of <140/90 mm Hg; [86] hence, we recommend that CKD patients without albuminuria be treated to maintain a BP that is consistently <140/90 mm Hg [87-89]. On the other hand, randomized controlled trials suggested that a lower target may be beneficial in proteinuric CKD patients. Thus, we recommend that CKD patients with albuminuria be treated to maintain a BP that is consistently <130/80 mm Hg [90-93]. BP target levels do not depend on the presence of DM [94].

Lifestyle modification should be used as a basic tool for BP control in all hypertensive CKD patients. Although no large scale randomized controlled trials have reported the effects of lifestyle modification on clinical outcomes in CKD patients, the beneficial effects can be inferred from the results reported in previous studies in general populations [95-101]. The 2007 Korea National Health and Nutrition Examination Survey showed that BMI and abdominal obesity were independently associated with the estimated glomerular filtration rate [102]. We recommend achieving or maintaining a healthy weight (BMI, 20 to 25), lowering salt intake to <90 mmol (<2 g) per day of sodium unless contraindicated, undertaking regular exercise compatible with CV health and tolerance, and limiting alcohol intake to <2 standard drinks per day for men and <1 standard drink per day for women.

Pharmacologic treatment in CKD patients includes single or multiple antihypertensive therapies to achieve the target BP. Although any antihypertensive can be used in CKD patients, ACE inhibitors or ARBs have been reported to be renoprotective owing to the reduction in proteinuria and improvement in the rate of decline in glomerular filtration rate [93,103-105]. Thus, as shown in Table 1, we recommend that an ACE inhibitor or ARB be used in CKD patients with albuminuria. ACE inhibitors or ARBs are preferred in both diabetic and non-diabetic CKD patients with either microalbuminuria (range, 30 to 300 mg/day) or macroalbuminuria (>300 mg/day).

**Table 1:** **Target blood pressure and preferred drugs in hypertension of adult chronic kidney disease patients**

**Albuminuria**	**Blood pressure target (mm Hg)**	**Preferred drugs**
No (<30 mg/day)	<140/90	None
Yes (≥30 mg/day)	<130/80	Angiotensin-converting enzyme inhibitors or angiotensin receptor blockers

It should be noted that there are occasions when the BP target and the preferred agent mentioned above may be inappropriate. Treatment should be individualized based on the patient’s age, presence of albuminuria, and comorbidities. Diabetic or elderly patients need to be asked about orthostatic dizziness because of the possibility of postural hypotension [106-108]. ACE inhibitors and ARBs are contraindicated in bilateral renal artery stenosis and should be used with caution in patients with diffuse atherosclerosis.

### Cerebrovascular diseases

The risk of ischemic and hemorrhagic stroke increases proportionally as the BP increases, with HTN being the most common adjustable risk factor in stroke prevention and having the highest attributable risk for stroke in the population. HTN treatment, particularly SBP control, will remarkably reduce the incidence of stroke. For the management of high BP, lifestyle modification (weight loss, low-fat diet, reduced salt intake, exercise or physical activity, moderation of alcohol intake, and smoking cessation) must routinely precede drug therapy. According to an epidemiologic study, with each increase of 20/10 mm Hg for BP > 115/75 mm Hg, deaths from stroke increased at least twofold. Conversely, a 10/5 mm Hg decrease in BP resulted in a 40% decrease in deaths from stroke [41]. In addition, a meta-analysis of clinical studies showed that the stroke risk was expected to decrease by about 30% to 40% by lowering the BP by 10/5 mm Hg with drug therapy, regardless of the past history of the patient [43,109,110]. For the primary prevention of stroke, it is recommended to maintain the BP <140/90 mm Hg [84,111]. Although it remains unknown whether a specific drug or class is superior to other antihypertensive drugs in the prevention of stroke, a limited number of reports show that calcium antagonists, ACE inhibitors, or ARBs are superior to beta-blockers [112]. However, for the primary prevention of stroke, it is most important to lower the BP based on an individualized approach for each patient rather than the choice of a specific drug or class of drug [113].

#### Acute ischemic stroke

In general, the BP rises in acute ischemic stroke. It is assumed that BP increases owing to the acute stress, previous HTN, and the automatic compensation in an attempt to maintain perfusion of the brain tissue in the ischemic state [114]. Thus, continuous BP monitoring is important, as a sudden drop in BP should be avoided to maintain the appropriate perfusion to the brain. Although a previous study has shown that ARB administration for 1 week in stroke patients within a week of the attack reduced the mortality after a 12-month period, [25] large-scale studies are needed to resolve the debates [115]. On the contrary, because active treatment against rising BP would reduce the perfusion to the ischemic area and expand the area of the infarction, it is undesirable to lower the BP actively within the period of 1 week of the acute ischemic stroke [18,116].

When thrombolytic therapy is used in the hyperacute period of an ischemic stroke, the incidence of bleeding is closely related to the BP before and after thrombolysis; hence, the target BP should be <185/110 mm Hg. For thrombolytic therapy using t-PA, the drug could only be administered after the BP was <185/110 mm Hg. The antihypertensive regimens consisting of intravenous drugs such as labetalol, nicardipine, diltiazem, nitroglycerin, and nitroprusside are recommended [117-120].

In the acute phase of the ischemic stroke, it is recommended to use antihypertensive drugs only when BP is >220/120 mm Hg, to avoid a decrease in the cerebral perfusion around the infarct area [121]. Target BP levels should be 85% to 90% of the baseline BP. However, in cases of hypertensive encephalopathy, aortic dissection, acute renal failure, acute pulmonary edema, and acute myocardial infarction, it is recommended to lower the BP sufficiently in order to prevent complications associated with the elevated BP itself [117,118].

#### Acute parenchymal hemorrhage

From a theoretical viewpoint, the optimal BP treatment in the acute phase of a parenchymal hemorrhage could prevent re-bleeding and subsequent expansion of a hematoma and edema; hence, it is recommended to lower the BP during the acute phase of the hemorrhage. If the SBP is ≥200 mm Hg or if the mean BP is ≥150 mm Hg, the BP should be lowered by monitoring BP every 5 min. In patients with increased intracranial pressure, BP should be lowered by maintaining the cerebral perfusion pressure between 60 to 80 mm Hg using an intracranial pressure monitoring device only when the SBP is >180 mm Hg or the mean BP is >30 mm Hg. Because a sudden drop of BP during the acute phase is associated with higher mortality rates, it is recommended to maintain a cerebral perfusion pressure of ≥60 mm Hg. When the SBP is 180 mm Hg or the mean BP is 130 mm Hg—as long as there is no evidence of increased intracranial pressure, with the assessment of BP every 15 min—the BP should be lowered below the level of 80% of baseline BP, 160 mm Hg in SBP and 90 mm Hg in DBP, or 110 mm Hg in the mean BP. The antihypertensive regimens including intravenous drugs such as labetalol, nicardipine, diltiazem, nitroglycerin, and nitroprusside are recommended [122]. Despite a recent report on benefits associated with lowering the BP to 140 mm Hg during the acute phase, the supportive evidence is insufficient; hence, the BP should be gradually and cautiously reduced [123,124].

#### Secondary prevention of stroke

HTN treatment as a measure of secondary prevention after stroke significantly reduces mortality and the recurrence of stroke or vascular diseases [109,125,126]. Regardless of the history of HTN, treatment of HTN after stroke significantly reduces the mortality and complications associated with HTN. Lifestyle modifications should be maintained in addition to the pharmacologic treatment. For selection of the optimal antihypertensive drugs, the individual characteristics of the patient such as the presence of extracranial cerebrovascular disease, kidney disease, heart disease, and diabetes should be considered. In a recent meta-analysis, a combination therapy using ACE inhibitors and diuretics was preferred [127].

### Aortic disease

In patients with aortic aneurysm, it is strongly recommended to lower the BP to the lowest levels tolerated by the patient [128]. Beta-blockers are preferred owing to their ability to reduce the maximum ventricular ejection of the left ventricle, as well as the BP and heart rate, but there is no controlled study for non-Marfan origin [129]. In the acute aortic syndrome, including aortic dissection, the BP and heart rate should be controlled aggressively with a regimen that includes a beta-blocker [130].

### Erectile dysfunction

Erectile dysfunction in hypertensive patients is considered to be one of the CV risk factors [131] associated with poor prognosis. Therefore, risk factors such as DM, dyslipidemia, and smoking should be controlled aggressively, and lifestyle modifications should be suggested to patients to reduce the CV risk [132]. However, most erectile dysfunction cases in hypertensive patients are not diagnosed by a doctor, and only a minority of patients seeks medical advice. Therefore, in order to improve CV prognosis with regard to erectile dysfunction, more cautious history taking is required to ensure it is incorporated in clinical decision making [133].

In general, the prevalence of erectile dysfunction in patients with HTN is reported to be 0% to 25%, but its evaluation is difficult because of the impact of the underlying diseases. Erectile dysfunction related to the administration of a specific drug is usually observed within 4 weeks. If the association is clear, the drug could be replaced, but the possibility that the preexisting peripheral vascular disease is exaggerated should be kept in mind when the drug is replaced. Beta-blockers and diuretics are known to cause erectile dysfunction, and ACE inhibitors and calcium antagonists are neutral, whereas ARBs are sometimes reported to be beneficial [53].

With respect to the conventional beta-blockers administered for patients with erectile dysfunction, a vasodilating beta-blocker may be an alternative [53]. In patients with erectile dysfunction caused by antihypertensive drugs, the phosphodiesterase-5 (PDE5) inhibitor is relatively safe and effective and the additional BP lowering effect is negligible [133]. However, the BP after administration of PDE5 inhibitors should be clearly noted, and PDE5 inhibitors must not be coadministered with nitrates.

### Pregnancy

High BP occurring during pregnancy can be divided into four categories: 1) chronic HTN in pregnancy: existing HTN or taking antihypertensive medication before the 20th week of pregnancy, 2) gestational HTN: new HTN diagnosed after the 20th week of pregnancy in the absence of proteinuria, 3) preeclampsia: HTN diagnosed after 20 weeks of pregnancy accompanied by proteinuria (albumin more than 300 mg in 24-h urine or urine albumin/creatinine ratio of 300 mg/g or greater), and 4) preeclampsia superimposed on chronic HTN: preeclampsia is diagnosed in the chronic HTN in pregnancy. According to the level of BP, it is classified as mild: 140 to 149 mm Hg/90 to 99 mm Hg, moderate: 150 to 159 mm Hg/100 to 109 mm Hg, and severe: 160/110 mm Hg or higher.

Generally, there are few controversies about the drug treatment for BP of >160/110 mm Hg or higher. A study reported that a patient with BP of >150/95 mm Hg is more likely to be hospitalized due to incident stroke during the peripartum period [18,134]. In uncomplicated pregnancy, because there is no evidence, [135] BP is controlled below 150/100 mm Hg [136,137], but it is not recommended to lower DBP below 80 mm Hg [137,138].

Antihypertensive drugs used during pregnancy are methyldopa, labetalol, and nifedipine [139]. The specific drugs are selected in consideration of the class of drugs previously taken, their side effects, and the risk of teratogenicity. Because beta-blockers may cause fetal growth retardation, it is preferable to use beta-blockers later in the pregnancy. Diuretics should be prescribed cautiously because they can decrease the volume of water in the body. Because ACE inhibitors or angiotensin blockers may increase the risk of congenital malformations in pregnancy, it is recommended to replace those drugs before pregnancy or when planning a pregnancy. If a pregnancy is detected during the administration of ACE inhibitors or angiotensin receptor blockers, they should be discontinued and replaced promptly. In emergency situations, such as preeclampsia, intravenous labetalol is recommended, but intravenous nitroprusside or nitroglycerin could be alternatives. After delivery, BP should be controlled below 140/90 mm Hg.

Gestational HTN and preeclampsia are associated with a relatively high risk of developing HTN in the future, and preeclampsia is a risk factor for CVD. Patients with a history of preeclampsia have about a twofold risk for ischemic heart disease, stroke, and venous thrombosis [140] and a fourfold risk for developing sustained HTN [141]. Especially in case of preeclampsia within 32 weeks of gestation, stillbirth, and fetal growth retardation, the risk for HTN increases much more. Thus, for HTN during pregnancy, active BP control and lifestyle modification even after delivery is strongly recommended.

### Women and hypertension

In younger population, the woman has lower prevalence of HTN than the man. But after menopause, the prevalence of HTN in women increases rapidly that it catches up the prevalence in men in their 60s. And it becomes even higher than that in men in their 70s or 80s. As for age, the increase in pulse pressure is the same between men and women; however, the SBP and DBP are higher in women after menopause than before menopause. Care should be taken in the diagnosis of HTN in menopausal women, because white coat HTN occurs more frequently in these women.

After menopause, weight gain, hormonal changes, and psychological changes occur [142]. Especially, the deficiency of female hormones such as estrogen induces menopausal symptoms and many CV changes [143]. In the past, hormone replacement therapy (HRT) was widely recommended after menopause, but clinical studies found no preventive effects on CVD or it sometimes worsened, and therefore, HRT is no longer recommended for the purpose to prevent a CV event. Because HRT can increase BP, women who have a greater chance of developing HTN need to be carefully observed for a few months [144].

There is no difference in HTN treatment between women and men. Additionally, there is no difference in BP reduction and drug effects between women and men [145]. Oral contraceptives can increase BP in some subjects, but the effects are not severe and the occurrence of accelerated or malignant HTN is rare. Family history of HTN, past history of HTN during pregnancy, potential kidney disease, obesity, or a longer period of oral contraceptive use increase the risk. Therefore, in the early period of oral contraceptive use, BP needs to be carefully monitored, while periodic measurements are recommended thereafter.

## Abbreviations

ACE: Angiotensin-converting enzyme
ARB: Angiotensin receptor blocker
BMI: Body mass index
BP: Blood pressure
CKD: Chronic kidney disease
CV: Cardiovascular
CVD: Cardiovascular diseases
DBP: Diastolic blood pressure
DM: Diabetes mellitus
HDL: High density lipoprotein
HRT: Hormone replacement therapy
HTN: Hypertension
KNHANES: Korean National Health and Nutrition Examination Survey
LVH: Left ventricular hypertrophy
PDE5: Phosphodiesterase 5
SBP: Systolic blood pressure
UKPDS: United Kingdom Prospective Diabetes Study
